# Proportion of asylum seekers carrying multi-drug resistant microorganisms is persistently increased after arrival in the Netherlands

**DOI:** 10.1186/s13756-018-0455-5

**Published:** 2019-01-07

**Authors:** Sofanne J. Ravensbergen, Christina Louka, Alewijn Ott, John W. Rossen, Darren Cornish, Spyros Pournaras, Erik Bathoorn, Ymkje Stienstra

**Affiliations:** 1 University of Groningen, University Medical Center Groningen, Department of Internal Medicine/Infectious Diseases, Groningen, The Netherlands; 2 ESCMID study group for infections in travelers and migrants, Basel, Switzerland; 3 Department of Medical Microbiology, Certe, Groningen, The Netherlands; 4 University Medical Center Groningen, Department of Medical Microbiology and Infection Prevention, University of Groningen, Groningen, The Netherlands; 5 ESCMID study group for genomic and molecular diagnostics, Basel, Switzerland; 6 Babylon Primary Health Care Services, Elst, The Netherlands; 7 ‘ATTIKON’ University Hospital, Kapodistrian University of Athens, Athens, Greece

**Keywords:** Multidrug resistant organisms, MRSA, Asylum seekers, Carriage rate

## Abstract

**Background:**

Several studies have shown a high prevalence of multi-drug resistant organisms (MDRO) amongst asylum seekers when compared to the general population. The aim of this study is to assess the duration of MDRO carriage in this population.

**Methods:**

Data were retrospectively collected between January 1st 2014 through December 31st 2016. Study material included screening samples for MDRO carriage and clinical samples from asylum seekers in need of medical care. The study focused on methicillin-resistant *Staphylococcus aureus* (MRSA) and multi-drug resistant Enterobacteriaceae (MDRE). The rates of MRSA and MDRE detected were calculated every four weeks after arrival in the Netherlands.

**Results:**

Samples from 2091 asylum seekers were included. 1270 (60.7%) were female, median age was 26 years (IQR 20–34) and median number of days in the Netherlands until first sample was 67 (IQR 4–235). In the patients’ first obtained samples, the rate of MRSA varied between 4.5 and 13.0% per time interval after arrival. The rate of MDRE fluctuated between 7.4% and 25.0%. No particular decline in positivity rates in first obtained samples was observed after arrival in the Netherlands. In the group of asylum seekers who arrived more than one year ago, MRSA was isolated in a percentage of 5.1% (*n* = 273, median months after arrival 34.1 (IQR 16.5–63.1)) and MDRE in 9.4% (*n* = 276, median months after arrival 35.4 (IQR 17–65)).

**Conclusion:**

To our knowledge, this is the first study demonstrating that carriage rate of MDRO in asylum seekers remains high even after prolonged stay in the Netherlands. Longitudinal data on MDRO carriage after arrival in countries with a low MDRO prevalence are needed to determine optimal screening strategies, infection control measures and empirical antibiotic therapy.

## Background

During the last decade, millions of refugees have entered European grounds as a result of warfare, violence, political instability and poverty in several Asian, African and Middle Eastern areas. Furthermore, ongoing civil wars in Syria and Afghanistan have led to an unprecedented influx of refuge seekers in Europe [1]. Several studies have been conducted on carriage of multidrug-resistant organisms (MDRO) in asylum seekers. A recently published systematic review and meta-analysis on antimicrobial resistance amongst migrants in Europe, showed a pooled prevalence of any detected antimicrobial resistance (AMR) carriage or infection of 25.4% (95% CI 19.1–31.8). The pooled prevalence of methicillin-resistant *Staphylococcus aureus (*MRSA) was 7.8% (95% CI 4.8–10.7), and of multidrug-resistant Gram negative bacteria (MDRGN) 27.2% (95% CI 17.6–36.8) [2].

The high proportion of MDRO carriage among asylum seekers may have implications for countries with low MDRO prevalence like the Netherlands. Previously studied MDRO carriage rate amongst asylum seekers in need of medical care in the Netherlands was compared to the MDRO rate in the Dutch hospitalized population. A prevalence of 21.4% compared to 5.1% for multi-drug resistant Enterobacteriaceae (MDRE) and 9.7% versus 1.3% for MRSA in the asylum seekers population and the Dutch population was found, respectively. These findings support a policy of MDRO screening amongst asylum seekers in the Dutch setting [3].

The current Dutch protocol recommends screening of all asylum seekers for MRSA and MDRGN carriage at hospital admission or emergency care visit, regardless of other risk factors [4, 5]. The protocol does not provide information on the needs of screening in relation to the time that elapsed since arrival into the country. Information on duration of MDRO carriage is available for travelers. In a Dutch study, 83.2% of travelers who tested positive for MDRGN after visiting high prevalence countries, were spontaneously decolonized within 6 months after returning to the Netherlands [6]. The same pattern was observed in a German study in which 91.4% of returning travelers who tested positive for ESBL, were decolonized after 6 months [7]. Rational screening for MDRO in asylum seekers in the Netherlands can be improved based on information on the duration of MDRO carriage in asylum seekers after arrival in the Netherlands and can be more targeted based on risk factors for MDRO carriage within the group of asylum seekers.

In this retrospective study, we examine MDRO carriage among asylum seekers focusing on the time that elapsed since arrival in the Netherlands.

## Material and methods

### Asylum seeker procedure in the Netherlands and the Certe laboratory

Almost all asylum seekers arriving in the Netherlands start their asylum procedure at the national reception center for asylum seekers in the northeastern part of the Netherlands. Asylum seekers who are in need of (direct) medical care will report at the health care services in the asylum seeker center. The general practitioner of the health care services may refer the asylum seeker to a hospital in the area. More information on the asylum seeker procedure in the Netherlands can be found in a previously published paper [3]. The Certe laboratory performed all the routine microbiological diagnostics for this northeastern part of the Netherlands, including samples from eight hospitals, general practitioners, nursing homes and asylum seeker centers in the region.

### Study design

Data was retrospectively collected from the Certe laboratory and the national health care system for asylum seekers. Study material included screening samples for MDRO carriage before admission (throat, rectum, and nose) and clinical samples (e.g. blood, wounds, and urogenital) from asylum seekers. All of these samples were obtained as part of the standard care. EUCAST guidelines were used for susceptibility interpretation [8]. Patients who tested negatively during their first visit at the hospital, were retested on re-admission or when they visited the emergency department. Results from these samples between January 1st 2014 and December 31st 2016, were aggregated. We focused on MRSA, Vancomycin Resistant Enterococci (VRE) and multidrug-resistant Enterobacteriaceae (MDRE). In MDRE three resistance patterns were distinguished: Extended Spectrum Beta-Lactamase (ESBL)-production, Fluoroquinolone plus Aminoglycoside Resistant Enterobacteriaceae (QARE), Carbapenemase-Producing Enterobacteriaceae (CPE) [3].

### Selection of participants

All asylum seekers are registered on the asylum seeker center’s address. The postal codes referring to these addresses were used to identify patients as asylum seekers for the study. Demographic data such as age, sex, and date of sampling were collected from the laboratory system. Country of origin and arrival date in the Netherlands was documented using the health care system for asylum seekers. In case the arrival date was missing, the date of first visit to a general practitioner was used.

### Bacterial detection and analysis

#### MRSA

Screening samples were incubated on blood agar (Mediaproducts BV) for growth control, a selective Chrom ID MRSA (bioMérieux) and a Mueller Hinton broth with NaCl 2.5% (Mediaproducts BV). The selective broth was also subcultured on selective Chrom ID MRSA (bioMérieux) after one-night incubation. Growth of *S. aureus* was confirmed by Staphaurex Plus (Oxoid) and coagulase-test [3].

#### MDRE

The selective agar plates used for MDRE detection were McConkey with ciprofloxacin 0,5 mg/l and gentamicin 2 mg/l (Mediaproducts BV), a ChromID ESBL and a ChromID Carbapenemase agar (both bioMérieux). Presence of ESBL was confirmed with cefotaxime-clavulanate, ceftazidime- clavulanate and cefepime-clavulanate Etest strips (bioMérieux). Possible CPE was confirmed by CIM test and PCR and typed by the national reference network for CPE at the RIVM (National Institute for Public Health) [3].

#### VRE

For selective culture of Vancomycin Resistant Enterococcus faecalis or Enterococcus faecium (VRE,) rectal swabs were incubated for 2 nights at 35 °C in Brain Heart Infusion broth with 2% NaCl and 16 mg/l gentamicin plus 16 mg/l vancomycin. Subcultures were made on chromID VRE agar plate (bioMérieux). VRE confirmation was done by VanA/ VanB PCR using GeneXpert (Cepheid).

### Statistical analysis

Data was analyzed with SPSS Version 23.0. Descriptive statistics were used for the general characteristics and the duration of MDRO carriage. Data is presented as median with 25–75% inter quartile range (IQR) as appropriate. To study the carriage rate of MRSA and MDRE over time, all dates of first samples taken were compared to the arrival date in the Netherlands. Carriage duration through time was analyzed by four-weeks periods. Due to small population size the last period was defined as week 53 and after and is represented as one group. For determining MDRO carriage over time, we excluded all clinical samples, considering no antibiotic usage data were available.

## Results

### General characteristics

From January 2014 through December 2016, 2091 asylum seekers were tested for MDROs as part of their standard care. Screening and clinical samples were obtained based on the cause of visit/admission to the hospital and the anticipated causative agents leading to different number of asylum seekers tested for MRSA and MDRE. General characteristics of both study groups can be found in Table 1.

**Table 1:** General characteristics of the study population

Number of asylum seekers (*n* = 2091)		
Sex (female (%))	1270 (60.7)	
Age in years median (IQR^b^)	26 (20–34)	
Number of days in the Netherlands until first sample (IQR)	67 (4–235)	
	Number of asylum seekers	Number of MDRO positive asylum seekers (%)
Total number of asylum seekers tested for MRSA analysis	1954	185 (9.5)
Screening analysis	1777	159 (8.9)
Clinical analysis	177	26 (14.7)
Total number of asylum seekers tested for MDRE analysis	1789	331 (18.5)^a^
Screening analysis	1555	298 (19.2)
Clinical analysis	234	48 (20.5)

^a^ 15 positive asylum seekers had both for screening and clinical samples

^b^ *IQR* Interquartile range

**Fig. 1:**
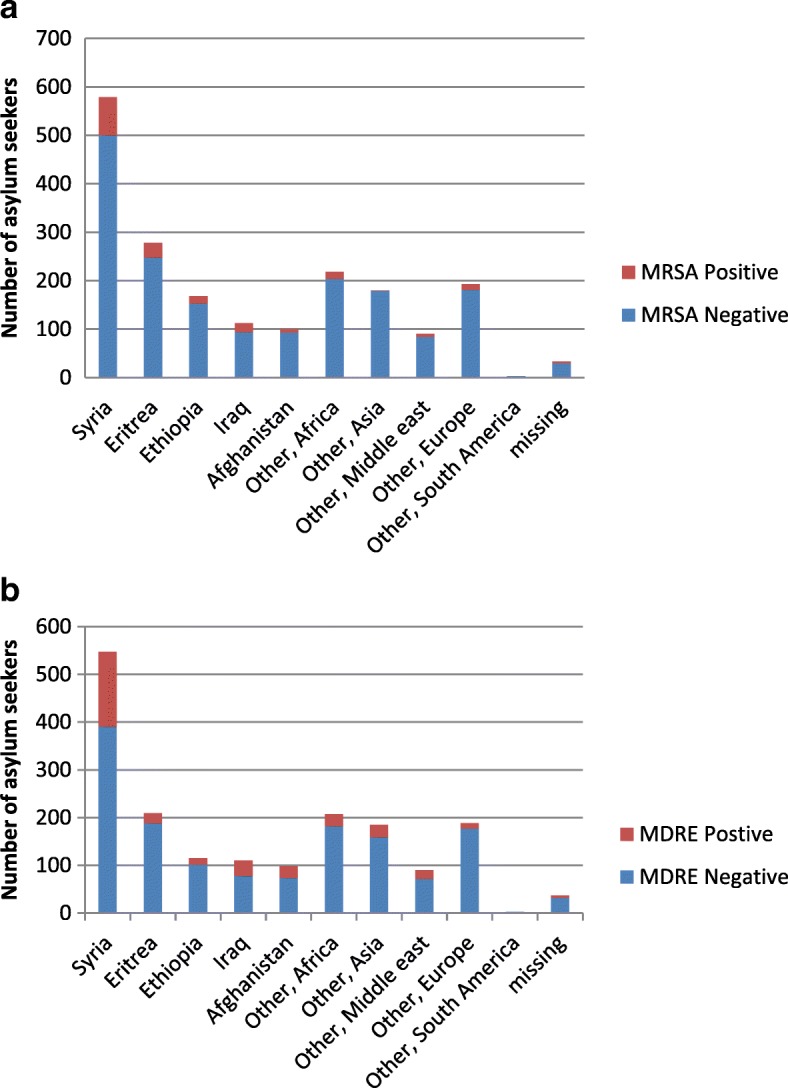
**a** Number of asylum seekers that tested positive for MRSA compared to the number of asylum seekers that tested negative for MRSA within country of origin. **b** Number of asylum seekers that tested positive for MDRE compared to the number of asylum seekers that tested negative for MDRE within country of origin

In total, 1954 asylum seekers were tested for MRSA, of which 185 (9.3%) were positive. Nine hundred seventy two asylum seekers were all tested negative for VRE. Furthermore, 1789 asylum seekers were tested for MDRE of which 331 (18.5%) tested positive. Specifics regarding screening to clinical samples ratio can be found in Table 1. MRSA and MDRE were relatively more frequently detected in asylum seekers originating from Iraq (19.1 and 43.2%, respectively) and Syria (15.8 and 39.9%, respectively). An overview of the country of origin of the study group and the number of people tested positive for MDRO within the total group, originating from the same country can be found in Fig. 1.

### Resistance patterns in MDRE

*E. coli* was the most frequently detected MDRE species (*n* = 301). The most frequent resistance pattern detected was ESBL-production followed by a combination of ESBL-production and Fluoroquinolone/Aminoglycoside resistance. Of note, one of the strains co-exhibited all three patterns of resistance. The carrier of this specific strain originated from Syria. Detailed numbers of species with specific resistance patterns can be found in Table 2.

**Table 2:** Number of isolates belonging to different microbial species and the respective resistance patterns they exhibited

Microbial species	ESBL^a^	QARE^b^	ESBL + QARE	CPE^c^ + ESBL	CPE + ESBL + QARE
Escherichia coli *n* = 301	240	12	48	1	0
Klebsiella pneumonia *n* = 26	19	0	7	0	0
Klebsiella oxytoca	0	0	1	0	1
Proteus mirabilis	5	2	0	0	0
Morganella morganii	0	0	2	0	0
Enterobacter cloacae	3	0	1	0	0
Citrobacter freundii	1	0	0	0	0
Total number of strains (*n* = 343^d^)	268	14	59	1	1

^a^ *ESBL* Extended Spectrum Beta-Lactamase

^b^ *QARE* Fluoroquinolone plus aminoglycoside resistant Enterobacteriaceae

^c^ *CPE* Carbapenemase-Producing Enterobacteriaceae

^d^ Total number of isolates (*n* = 343) is higher than the number of positive sample because same samples were positive for more than one MDRE

### MDRO carriage

#### MDRO carriage in the first culture

**Fig. 2:**
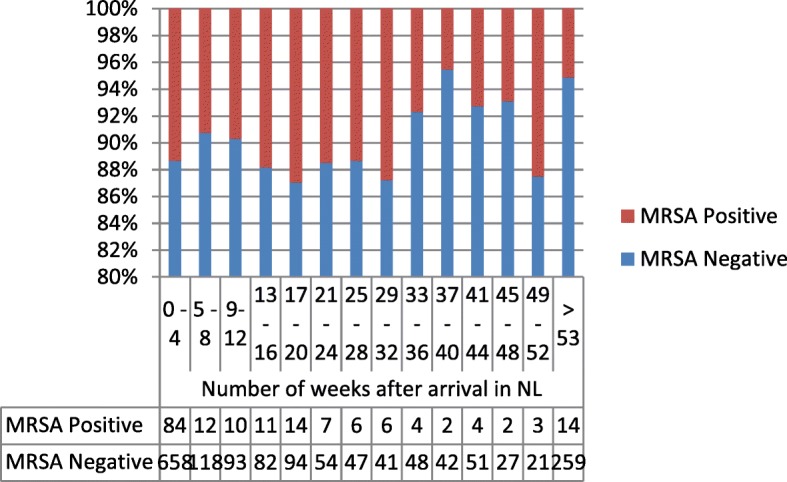
Percentage of MRSA in first obtained sample in relation to time (weeks) after arrival in the Netherlands. Repeated samples are excluded

**Fig. 3:**
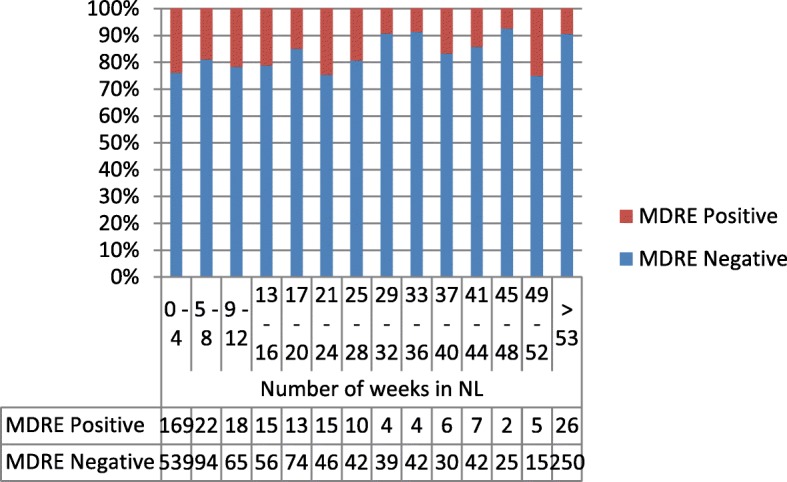
Percentage of MDRE in first obtained sample in relation to time (weeks) after arrival in the Netherlands. Repeated samples are excluded

Figures 2 and 3 show the carriage rate of MRSA and MDRE in asylum seekers in their first obtained sample in percentages over time. For MRSA, the median time after arrival in the last group (over 53 weeks) was 1022 days (IQR 494–1892) and for MDRE was 1063 days (IQR 510–1950).

#### MDRO carriage during follow-up

##### MRSA

Of the 1954 asylum seekers tested for MRSA, follow-up screening samples were performed in 442 of them after a median of 60 days (IQR 27–109) after the first sample was obtained. Forty seven asylum seekers that tested positive for MRSA, were followed up with screening samples. Twenty five of the asylum seekers who tested positive for MRSA in the first obtained sample, tested positive in their follow-up samples which were obtained after a median of 80 days (IQR 20.5–101).

Six of the asylum seekers who tested positive for MRSA in the first obtained sample, tested negative in the subsequent samples which were obtained after a median of 48.5 days (IQR 43.75–147). It is unknown if these patients received eradication or treatment before retesting.

There were 16 asylum seekers who initially tested negative for MRSA but tested positive for MRSA in repeated screening sample obtained after a median 63 days (IQR 8.5–109).

##### MDRE

Of the 1789 asylum seekers tested for MDRE, follow up screening samples were performed in 365 of them after a median of 57 days (IQR 27–104) after the first sample was obtained. Seventy-one of the asylum seekers who tested positive for MDRE, were followed up with screening samples. Thirty-eight of the asylum seekers who tested positive for MDRE in the first obtained sample, tested positive in their follow-up samples which were obtained after a median of 60.5 (IQR 16.8–99.8).

Twenty nine of the asylum seekers who tested positive for MDRE in the first obtained sample, tested negative in the subsequent samples which were taken after a median of 87 days (IQR 39–179). It is unknown if these patients received treatment before the retesting. There were four asylum seekers who initially tested negative for MDRE but tested positive for MDRE in repeated screening samples taken after a median of 28.5 days (IQR 5–163.8).

## Discussion

In this study, a large number of both screening and clinical samples were collected from 2091 asylum seekers. The most frequently isolated resistant microbes were MRSA and ESBL-producing *E. coli*. The percentage of MRSA and MDRE in asylum seekers who tested positive for MRSA and/or MDRE changed over time. However, no clear pattern of decline or increase was observed.

Several studies have described MDRO carriage or outbreaks amongst Syrian refugees. In a Swiss study, 261 refugees were screened for MRSA of which 41 (15.7%) tested positive. Furthermore 240 refugees were screened for ESBL-producing Gram negatives of which 57 (23.7%) tested positive [9]. In addition, studies in Germany have shown MDRO prevalence in asylum seekers ranging between 24.7% and 60.8%. The main resistant strains isolated were MRSA, and ESBL-producing Enterobacteriaceae [10, 11]. The wide range of carriage observed among these studies reflects the differences among the study population such as age, country of origin, included samples, e.g. screening and/or clinical, risk factors like previous hospitalization and differences in sampling strategies and laboratory methods. Our findings align with high MDRO range amongst this vulnerable population.

Studies have shown that the duration of MDRO colonization in humans vary across strains – e.g. the median clearance for MRSA varied between 5.9 and 9 months [12, 13]. Regarding ESBL-producing Enterobacteriaceae, a median clearance of 6.6 months was observed [14]. In an Australian study, 26 out of 48 (54%) international travelers cleared all resistant *E. coli* within 2 months after their return, while 18% remained colonized 6 months post-travel [15]. Furthermore, a study from the Netherlands, a country of low prevalence for MDROs, investigated MDRO carriage rate and its duration over time in travelers returning from high prevalence countries. One hundred thirteen people who were negative for MDRGN before travelling tested positive after returning to the Netherlands. 83.2% of them were spontaneously decolonized within 6 months after returning to the Netherlands [6].

We therefore expected the MDRO prevalence amongst our population group to decline over time, especially when taking under consideration the low prevalence of MDRO in the Netherlands. However, a significant percentage of our population group tested positive in their first sample taken even after more than 1 year after arrival in the Netherlands.

Multiple possible explanations for the prolonged duration of carriage of MDROs in this population can be considered. Firstly, a large number of the study group spent most of the study period living together in one of the asylum seeker centers in the northern part of the Netherlands. MDRO carriers could serve as a natural reservoir, forming a cluster of such strains and close contact within the facility could lead to transmission [16]. Secondly, the possibility of MDRO strains being part of the normal flora should be considered. In such cases, the resistant strains colonize the gut microbiome indefinitely and detecting them depends on the screening methods and their microbial load at the time of screening.

Due to the retrospective aspect of this study we did not have access to important information like traveling and antibiotic consumption history. However, our study population exhibited wide ranges regarding age, country of origin, and included both hospitalized and non-hospitalized asylum seekers. No systematic screening upon arrival in the Netherlands, and follow up screening was performed in asylum seekers. Only in asylum seekers in need of medical care multiple times or admitted to the wards, follow up screening was performed.

Antibiotic consumption in general is low in the Netherlands. The antibiotic consumption data by the study population is unknown to us, but a small percentage of the asylum seekers population face health issues such as infections and might have been treated with antibiotics. This could have contributed to emergence of resistant strains and/or prolonged duration of carriage due to antibiotic pressure.

In order to confirm or reject our hypothesis regarding prolonged duration of carriage, the next rational step would be to perform a prospective, longitudinal study of a cohort. However, it is unsure whether it could be perceived as ethical to approach asylum seekers for participation in a prospective trial upon arrival considering their dependent position. Moreover, molecular analysis of the strains and thorough examination of their phylogenetic relatedness could reveal important information on transmission and cluster formation.

## Conclusion

In conclusion, our findings verify the high MDRO prevalence among the asylum seeker population. To our knowledge, this is the first study demonstrating that carriage rate of MDRO remained high even after long term stay in the Netherlands. This finding has consequences for the optimal screening strategy, infection control measures and empirical antibiotic therapy. A prospective, longitudinal study of a cohort should be performed to confirm our findings. The dependent position of asylum seekers should however be carefully considered in the study design. Moreover, molecular analysis of the strains and thorough examination of their phylogenetic relatedness could reveal important information on transmission and cluster formation.
